# Mechanism of Mongolian Medicine *Eerdun Wurile* in Improving Postoperative Cognitive Dysfunction Through Activation of the PI3K Signaling Pathway

**DOI:** 10.3389/fnins.2021.769759

**Published:** 2022-01-14

**Authors:** Zhixin Lv, Limuge Che, Yiri Du, Jianshe Yu, Enboer Su, Hui Liu, Dongmei Chen

**Affiliations:** ^1^Department of Anesthesiology, The Affiliated Hospital of Inner Mongolia Medical University, Hohhot, China; ^2^Medical Innovation Center for Nationalities, Inner Mongolia Medical University, Hohhot, China

**Keywords:** postoperative cognitive dysfunction, hippocampal formation, signaling pathway, Mongolian medicine *Eerdun Wurile*, PI3K-AKT pathway

## Abstract

**Objective:**

To study the effect of *Eerdun Wurile (EW)*, a traditional Mongolian medicine, on the cognitive function of rats by activating the IRS-PI3K-AKT-GLUT4 pathway in an animal model of postoperative cognitive dysfunction (POCD).

**Methods:**

Fifty clean-grade adults Sprague Dawley (SD) male rats were assigned to one of five groups: (1) a control group with no anesthesia (Group C), (2) a POCD model group with anesthesia only (Group P), (3) POCD group with low-dose EW treated (Group L), (4) a POCD group with high-dose EW treated (Group H), and (5) a POCD model group with dexmedetomidine treated (Group D) for positive control. The study started 7 days after all rats had acclimated to housing. Rats were trained in the Morris Water Maze navigation 5 days before surgery. All rats underwent the same maze for navigation and spatial exploration experiments on the preoperative day 1 and postoperative days 1, 3, 5, and their learning and memory abilities were assessed. At the end of the water maze experiment, rats were sacrificed to obtain hippocampal tissue. The mRNA levels of IRS-2, PI3K, AKT, and GLUT4 were measured in the hippocampus by real-time PCR, and the expression of IRS-2, PI3K, AKT, and GLUT4 protein in the hippocampus was determined by Western blotting to investigate the potential mechanisms at the molecular level.

**Results:**

Compared to control Group C, Group P, L, H, and D showed prolonged escape latency (*P* < 0.05) and decreased number of times to cross the platform (*P* < 0.05) at 1, 3 and 5 days after surgery. Compared to Group P, Group L, H, and D showed a decrease in escape latency with an increased number of crossing the platform at all-time points after surgery (*P* < 0.05). Within individual P, L, H, and D groups, escape latencies decreased (*P* < 0.05) and the number of times that the platform was crossed increased (*P* < 0.05) between postoperative days 3 and 5 compared to postoperative 1 day. Compared to Group C, the mRNA expression of IRS-2, PI3K, AKT and GLUT4 in the hippocampus of P, L, H, and D groups were decreased (*P* < 0.05). Compared to Group P, IRS-2, PI3K, AKT, and GLUT4 in the hippocampus of L, H, and D groups were increased (*P* < 0.05). Compared with Group D, the expression levels of IRS-2 and AKT in both L and H groups were higher. The expression level of PI3K in Group L was also higher (*P* < 0.05) vs Group D. The expression of AKT mRNA in Group H was higher than in Group L (*P* < 0.05). Compared to Group C, the p-IRS-2/IRS-2 ratio in the hippocampus of Group P was higher than that of Group C (*P* < 0.05). Compared to Group P, the ratios of p-IRS-2/IRS-2 in Group L, Group H, and Group D were lower, and the ratios of the p-PI3K/PI3K, p-AKT/AKT, and p-GLUT4/GLUT4 were higher (*P* < 0.05).

**Conclusion:**

Administration of EW showed the effect on the signaling pathway in rats with POCD. The therapeutic effect was better in the low-dose group. This could be related to the insulin downstream signal molecule PI3K and the IRS-PI3K-AKT-GLUT4 signaling pathway.

## Introduction

Postoperative cognitive dysfunction (POCD) is a global public health issue with an aging population that needs to be urgently solved. A total of 9–54% of adults over 65 years of age have been reported to develop POCD within 1 week after surgery (Androsova et al., 2015). As the population ages in China and the number of critically ill patients undergoing surgeries increases, the yearly incidence of POCD may increase. This will have a negative impact on the quality of life in older patients (Du and Weidong, 2019) and on social resources. The Mongolian medicine Eerdun Wurile (EW) produces apparent therapeutic effects on memory deterioration, cognitive decline, nerve palsy, and cerebral spinal cord damage. EW has been used to treat Sa disease (a cerebrovascular disease characterized by sudden dizziness, hemiplegia and unclear speech) along with Zachong-13 by Shan and Xia (2016), and has presented a satisfactory effect on symptoms of retardation of expression, memory deterioration, dizziness, and drowsiness. Furthermore, EW can significantly improve the neurobehavioral function of rats with a middle artery obstruction/reperfusion injury, narrow cerebral infarction area, and alleviate cerebral edema (Lian et al., 2014). Meanwhile, it also inhibits cell necrosis in the brain prefrontal cortex (Lian et al., 2016), and suppresses nerve cell apoptosis caused by ischemia and hypoxia injury in rat brain tissues (Chun et al., 2013). Preliminary results of this project have demonstrated abnormal glucose metabolism in the brain of adult POCD rats. As the insulin signaling pathway genes IRS-2, PI3K, AKT, and GLUT4, which are intimately related to glucose metabolism, have been down-regulated, this signaling pathway is considered involved in the POCD process (Du et al., 2019). Therefore, we hypothesized that EW would have a therapeutic effect on POCD via its potential regulatory mechanism on abnormal glucose metabolism.

## Materials and Methods

### Animal Grouping

A total of 50 clean-grade adult male Sprague Dawley (SD) rats aged 10–12 months, weighing approximately 500 g, were provided by Beijing Xinglong Experimental Animals Co., Ltd. The rats were divided into five groups (*n* = 10) using a random number table method, including a normal control group (Group C) without anesthesia, a POCD model group without any treatments (Group P), a low-dose EW group (Group L), a high dose EW group (Group H), and a dexmedetomidine group (Group D) as a treatment control group. All the rats were raised in a clean environment with 12 h light and dark alternatives. Food and water were available at will. The breeding room temperature was set at 20–24°C. The experiment was started after 7 days of adaptive feeding. The specific grouping method was as follows:

1) Group C: rats were not given special treatment, except an equal amount of distilled water was administered by gavage.
2) Group P: an equal amount of distilled water was administered before surgery by gavage. One hour before surgery, the rats were intraperitoneally injected with 100 μg/kg lipopolysaccharide (LPS), anesthetized using 10% chloral hydrate (0.3 mL/100 g), and a left nephrectomy was performed.
3) Group L: low-dose EW (0.63 g/kg) was administered by continuous intragastric administration 3 days before surgery. One hour before surgery, the rats were intraperitoneally injected with 100 μg/kg LPS, anesthetized using 10% chloral hydrate (0.3 mL/100 g), and a left nephrectomy was performed. Low-dose EW was administered by gavage 2 h after surgery, and an equal volume was administered by gavage daily until the 5th day after surgery.
4) Group H: high dose EW (1.26 g/kg) was administered by continuous intragastric administration 3 days before surgery. One hour before surgery, rats were peritoneally injected with 100 μg/kg LPS, anesthetized using 10% chloral hydrate (0.3 mL/100 g), and a left nephrectomy was performed. High dose EW was administered by gavage 2 h after surgery, and an equal volume was administered by gavage daily until the 5th day after surgery.
5) Group D: rats were injected with 100 μg/kg LPS (IP, 1 h before the surgery) and 25 μg/kg dexmedetomidine hydrochloride (IP, 15 min before surgery) and then anesthetized using 10% chloral hydrate (0.3 mL/100 g), followed by left nephrectomy. The oral dose of EW for an adult is 13–15 granules once or twice a day, 2 g/10 granules, approximately 3 g/day.

We used this weighted-based dosing for rats. Rats were administered intragastrically at a dose per kilogram of body weight (low-dose administration: 0.63 g/kg and high dose 1.26 g/kg).

### Behavioral Experiments

The Morris Water Maze included a cylindrical water tank as a hidden platform for animals to inhabit. The tank was divided into four quadrants: the first, second, third, and fourth quadrants. Each had a fixed place for the experimental animal and a heater to maintain the water temperature at around 24°C. The hidden habitat platform was 1 cm below the water level and 10 cm in diameter, placed in a certain quadrant for laboratory animals to find. A video analyzer was used to record the tracks, swimming speed, escape latency, and the number of crossings of the platforms of the animals in the water maze. All experimental rats were trained in positioning and navigation 5 days before surgery. Meanwhile, the video analysis system analyzed the running track and recorded relevant data, namely escape latency. All rats were placed in a randomly selected quadrant facing the wall 1 day before surgery, then 1, 2, 3, and 5 days after surgery (POD 1, POD 3, and POD 5). The swimming speed, the number of crossings in each quadrant, and the escape latency were recorded. Subsequently, the hidden platform was removed, a quadrant was randomly selected again, and the rats were placed in a water maze tank facing the wall for spatial exploration tests. The number of platform crossings within 60 s was recorded (Chong et al., 2016).

### Modeling and Material Selection

Except for Group C, all experimental rats of Group P, L, H, and D fasted for 12 h before surgery and intraperitoneally injected LPS 100 μg/kg 1 h before surgery. Additionally, five min before surgery, the animals were intraperitoneally injected with 0.3 mL/100 g 10% chloral hydrate mixture as anesthesia. When the rats lost their reflex, the fur under the left costal margin was shaved to prepare the skin. The animal was fixed on the operating bench in a right lateral position. Routine iodophor disinfection was performed twice before surgery, and sterile towels were spread. A longitudinal incision of 2–3 cm in length was made along the midaxillary line under the left costal margin. The abdominal cavity was dissected to fully expose the renal pedicle. After ligation of the ureter, renal artery, and renal vein at a distance of 0.5 cm from the renal hilum, the left kidney was removed. When the properpro reflex was regained, the rats were delivered to the breeding house to continue feeding. Combined with water maze tests and related laboratory examinations, the occurrence of POCD after surgery was determined. On POD 5, when all the rats received and completed behavioral experiments, they were anesthetized with 10% chloral hydrate, and the decapitated and hippocampal tissues were subsequently removed. Real-time fluorescence quantitative PCR assay was used to determine the mRNA expression of the relevant factors, and the operating procedures followed the instructions of the kit (Tiangen Biotech Co., Ltd.). The Ct value of each sample was obtained using the real-time quantitative PCR technique. The primer sequences are shown in Table 1.

**TABLE 1:** Primer sequences.

Gene	Forward primers (5′-3′)	Reverse primers (5′-3′)
IRS-2	TGAGAGCGAGAAGAAGTGGAAG	CTTGGTGTAGAGGGCGATCAG
PI3K	GTCGTTGATAGACCACCGCTTCC	TGCCCTGTTCCTCTGCCTTCC
AKT	CAAGCACCGTGTGACCATGA	TCAGTAAGCGTGTGGGCAAC
GLUT4	CCAGTATGTTGCGGATGCTATG	GAAGGTGAAGATGAAGAAGCCAAG
β-actin	GTGCTATGTTGCTCTAGACTTCG	ATGCCACAGGATTCCATACC

Western blot detection was used to determine the protein expression levels of the relevant factors. The tissues were lysed with RIPA lysis buffer for protein extraction. After the RIPA lysate was dissolved, PMSF was added at the volume ratio of RIPA:PMSF = 100:1, and placed on ice for subsequent use. After lysing the tissue lysate in an ice bath for 30 min, centrifugation was performed at 12,000 rpm 4°C for 15 min and stored at −20°C or −80°C for later use after the supernatant. The BCA method was used to determine the protein concentration. The samples were added with a 5× loading buffer and boiled for eight min. Proteins were separated by SDS-PAGE gel electrophoresis, transferred to the NC membrane using a wet transfer method, and blocked with 5% skim milk at room temperature for 1 h. The primary antibody was used with the primary antibody diluent according to the described ratios: IRS-2 (1:1 000, Cell Signaling Technology, United States), PI3K (1:1 000, Cell Signaling Technology, United States), AKT (1:1 000, Cell Signaling Technology, United States), and GLUT4 (1:1 000, Proteintech, United States). The primary antibody was supplemented and incubated overnight at 4°C. The primary antibody was then collected, washed twice using an appropriate amount of 1× TBST, 8 min each time, and washed twice again with an appropriate amount of TBS, 5 min each time. After secondary antibody incubation and washing, TBS was removed, and the NC membrane was quickly transferred to an EP tube with secondary antibody diluted at 1:1 000 and incubated at room temperature for 1 h. The NC membrane was scanned using an infrared fluorescence analyzer, and the supporting software was utilized to analyze the gray value of the protein bands. The gray value ratio of the target band to the internal reference band was used as the expression level of the target protein in each group. Each sample was analyzed three times, and the average and standard deviation were calculated.

### Statistical Methods

SPSS 22.0 software was used for statistical analysis. The measurement data were tested for normality and homogeneity of the variance, and the normal distribution was expressed as mean ± SD (*x* ± *s*). One-way analysis of variance was used for comparison among groups, and analysis of variance with repeated measurement design was used to compare different time points within groups. *P* < 0.05 was considered statistically significant.

## Results

**FIGURE 1:**
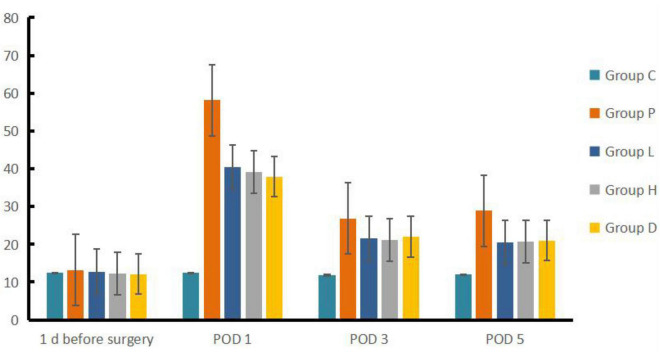
Comparison of escape latency among the five groups.

**FIGURE 2:**
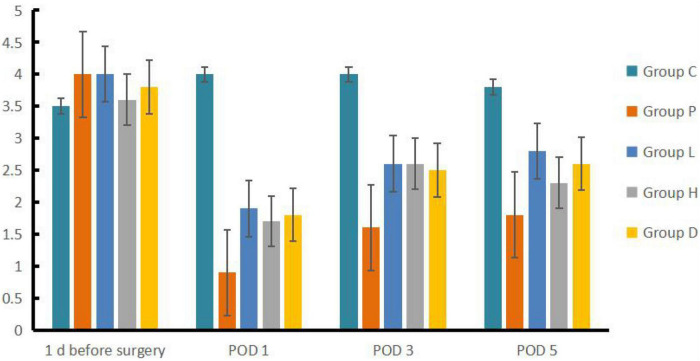
Comparison of the number of crossing platforms among the five groups.

Compared to Group C, Group P, L, H, and D had a prolonged escape latency at POD 1, 3 and 5 (*P* < 0.05), and the number of crossing platforms decreased (*P* < 0.05). Compared to Group P, Group H, L, and D showed reduced escape latency but an increased number of platform crossings at each time point after surgery (*P* < 0.05). Compared to Group P, Group L, H, and D escape latency was lower at POD 3 and POD 5 than on POD 1 (*P* < 0.05), whereas the number of crossing platforms increased (*P* < 0.05) (see Figures 1, 2).

**FIGURE 3:**
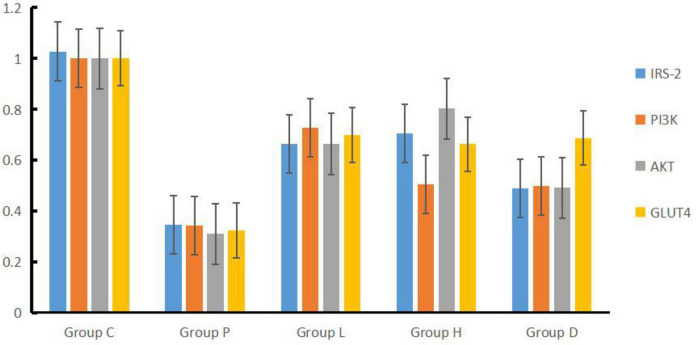
Concentrations of IRS-2, PI3K, AKT, and GLUT4 factors in hippocampal tissues among the five groups.

Compared to Group C, the expressions of IRS-2, PI3K, AKT, and GLUT4 genes mRNA in the hippocampal tissues of Group P, H, L, and D decreased (*P* < 0.05). On the other hand, and compared with Group P, the mRNA expressions of the four genes described in Group H, L, and D increased (*P* < 0.05). In addition, compared with Group D, IRS-2 and AKT mRNA expression levels in Group L were higher, PI3K mRNA expression levels in Group L were higher than in Group D (*P* < 0.05), and mRNA expression of AKT in Group H was higher than that in Group L (*P* < 0.05), see Table 2 and Figure 3.

**TABLE 2:** Comparison of mRNA levels of IRS-2, PI3K, AKT, and GLUT4 genes in hippocampal tissues the five groups (*n* = 10, *x* ± *s*).

	Group C	Group P	Group L	Group H	Group D
IRS-2	1.028 ± 0.291	0.347 ± 0.0716^a^	0.665 ± 0.078abd	0.706 ± 0.109^ab^	0.489 ± 0.06^abc^
PI3K	1.001 ± 0.049	0.342 ± 0.086^a^	0.729 ± 0.109abcd	0.505 ± 0.114^ab^	0.498 ± 0.084^ab^
AKT	1.0 ± 0.035	0.31 ± 0.063^a^	0.664 ± 0.175abcd	0.803 ± 0.168^ab^	0.491 ± 0.083^abc^
GLUT4	1.002 ± 0.077	0.323 ± 0.06^a^	0.699 ± 0.094ab	0.663 ± 0.153^ab^	0.688 ± 0.073^ab^

*A represented P < 0.05 compared to Group C; b represented p < 0.05 compared to Group P. c represented P < 0.05 compared to Group H; d represented P < 0.05 compared to Group D.*

**FIGURE 4:**
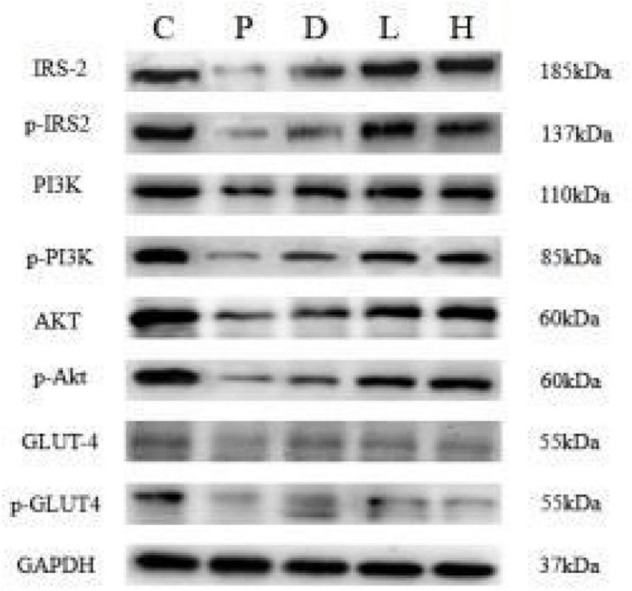
Comparison of expressions of each factor in hippocampal tissues among five groups.

Compared to Group C, the p-IRS-2/IRS-2 ratio of the hippocampal tissues in Group P was higher than that of the control group, and the difference was statistically significant (*P* < 0.05). Meanwhile, compared to Group P, the P-IRS-2/IRS-2 ratio in Groups H, L, and D was low, while the p-PI3K/PI3K, p-AKT/AKT ratios p-GLUT4/GUT4 genes increased (*P* < 0.05), as shown in Figure 4.

## Discussion

Based on the results of the water maze, we found that the rats in Groups P, L, H, and D had statistically significantly longer escape latency in POD 1, 3, and 5, along with a reduction in the number of crossing platforms compared to both 1 day before surgery by their own groups and Group C at each studied time after surgery, indicating that the establishment of the model was successful. We did find that EW has effects on POCD in rat model insults from both exploration anesthesia and left nephrectomy in Group L and H, similar to the effect of Group D as the therapeutic control of dexmedetomidine, and better than the non-treatment Group P in both latency and the number of crossing platforms compared to both 1 day before surgery by their own groups and Group P at every studied time after surgery. However, the effects did not appear to be dosing dependent given the fact that the number of crossing platforms in Group L was higher than that in Group H in POD 5. Furthermore, as a positive control group, D may have a different pathway than Group H and L, as the mRNA expressions of the four genes were lower than those of Group L and H.

The specific mechanism of POCD pathogenesis remains unclear and is probably multifactorial. Normal glucose metabolism is known to lead to a series of vascular and neurodegenerations that impair cognitive function. In the case of insulin-induced severe hypoglycemia, the brain glucose level rapidly descends, leading to cognitive dysfunction, namely seizure and coma (Nacca et al., 2018). Alternatively, hyperglycemia can damage cerebral vascular tissues by injuring blood vessels, altering metabolism and neuronal calcium homeostasis, and causing changes and inflammatory reactions. This can lead to cognitive dysfunction and problems with the processing and integration of information, leading to impaired cognitive response and processing capacity (Feinkohl et al., 2019). Additional studies have shown that intraperitoneal injection of LPS can cause severe central nervous system degeneration (Fidalgo et al., 2011). However, some recently published systematic reviews and meta-analyses have shown that metabolic disorders in patients, including elevated blood glucose, blood pressure, and obesity, are closely correlated with the appearance and development of POCD (Hovaguimian et al., 2018). As shown previously, PET/CT detected abnormal glucose metabolism in the brain of adult POCD rats, further confirming that POCD was related to the glucose metabolism disorder in the brain (Du et al., 2019). Furthermore, abnormal energy metabolism is the main pathological cause of the occurrence and development of neurocognitive dysfunction after surgery. Information on the selection of lipopolysaccharide dosage, surgical model, and dosage can be found in the literature (Wang, 2016).

The hippocampal tissue factors have shown a similar pattern indicating these factors would be highly linked to the data shown in the water maze results as one of the potential causal effects or mechanisms. The results of quantitative fluorescence PCR indicated that the mRNA expressions of the IRS-2, PI3K, AKT, and GLUT4 genes in the hippocampal tissues of Group P, L, H, and D decreased compared to Group C. Compared to Group P, all the expressions of the four described genes in Groups L, H, and D were elevated. The results revealed that the abnormality of insulin signaling pathway IRS-PI3K-AKT-GLUT4 at the genetic level might be the mechanism of POCD-induced cerebral glucose metabolism disorder caused by surgery combined with peripheral inflammation. Western blot findings were consistent with the expressions at the genetic level, which further verified that the insulin signaling pathway in the hippocampal tissues of POCD rats was disordered and glucose metabolism abnormal, consistent with the finding of the maze data.

The study has certain limitations. First, since chloral hydrate has no analgesic effect, anesthesia with it has the potential to interfere with the outcome of this experiment and is inhumane to experimental animals. Later, we will further standardize the use of anesthetics to establish a more regulated model to observe the effects of different anesthetics on the results of this study. Second, this experiment explains the changes in human body mechanisms from the perspective of animals, but there are still differences between animals and humans. There is still a long way to go for this study to be applied to clinical practice in the future. Third, we are uncertain whether EW acts directly or indirectly on this pathway to exert its effect, and further studies are needed in the future. Furthermore, in the escape study, all treated groups (except for Group C) had varying levels of latency. Except for the reason of POCD, it could be due to postoperative pain. In Groups H, D, and L, the medicine is given may have different levels of analgesic functions. However, studies on postoperative analgesia by EW are rarely reported.

This experiment is the first step taken by our research group on the impact of EW on POCD. There are many problems to be solved. For example, we are not sure why the effect of the low-dose group is better than that of the high-dose group, and what role does the concentration of EW play? Also, will anesthesia with chloral hydrate affect the results of this experiment? This needs to be confirmed by further research in the future.

Taken together, changes in this signaling pathway were observed in rats with POCD. And the low-dose group could be more useful in the treatment of POCD. The mechanism may be correlated with the downstream insulin signaling molecule PI3K and the IRS-PI3K-AKT-GLUT4 signaling pathway involved in its regulation.

## Data Availability Statement

The original contributions presented in the study are included in the article/Supplementary Material, further inquiries can be directed to the corresponding author.

## Ethics Statement

The studies involving animals were reviewed and approved by the Medical Ethics Committee of Inner Mongolia Medical University No. YKD202101074.

## Author Contributions

YD, JY, DC, and LC designed and supervised the whole experimental process. ES, HL, and ZL performed the experiments. ZL and LC provided technical assistance and statistical analysis and reviewed and edited the manuscript. All authors approved the final version of the manuscript.

## Conflict of Interest

The authors declare that the research was conducted in the absence of any commercial or financial relationships that could be construed as a potential conflict of interest.

## Publisher’s Note

All claims expressed in this article are solely those of the authors and do not necessarily represent those of their affiliated organizations, or those of the publisher, the editors and the reviewers. Any product that may be evaluated in this article, or claim that may be made by its manufacturer, is not guaranteed or endorsed by the publisher.
